# Ruxolitinib synergizes with DMF to kill *via* BIM+BAD-induced mitochondrial dysfunction and *via* reduced SOD2/TRX expression and ROS

**DOI:** 10.18632/oncotarget.8039

**Published:** 2016-03-15

**Authors:** Mehrad Tavallai, Laurence Booth, Jane L. Roberts, William P. McGuire, Andrew Poklepovic, Paul Dent

**Affiliations:** ^1^ Department of Biochemistry and Molecular Biology, Virginia Commonwealth University, Richmond, VA, USA; ^2^ Department of Medicine, Virginia Commonwealth University, Richmond, VA, USA

**Keywords:** ruxolitinib, JAK-STAT, DMF, tecfidera, ROS

## Abstract

We determined whether the myelofibrosis drug ruxolitinib, an inhibitor of Janus kinases 1/2 (JAK1 and JAK2), could interact with the multiple sclerosis drug dimethyl-fumarate (DMF) to kill tumor cells; studies used the in vivo active form of the drug, mono-methyl fumarate (MMF). Ruxolitinib interacted with MMF to kill brain, breast, lung and ovarian cancer cells, and enhanced the lethality of standard of care therapies such as paclitaxel and temozolomide. MMF also interacted with other FDA approved drugs to kill tumor cells including Celebrex^®^ and Gilenya^®^. The combination of [ruxolitinib + MMF] inactivated ERK1/2, AKT, STAT3 and STAT5; reduced expression of MCL-1, BCL-XL, SOD2 and TRX; increased BIM expression; decreased BAD S112 S136 phosphorylation; and enhanced pro-caspase 3 cleavage. Expression of activated forms of STAT3, MEK1 or AKT each significantly reduced drug combination lethality; prevented BAD S112 S136 dephosphorylation and decreased BIM expression; and preserved TRX, SOD2, MCL-1 and BCL-XL expression. The drug combination increased the levels of reactive oxygen species in cells, and over-expression of TRX or SOD2 prevented drug combination tumor cell killing. Over-expression of BCL-XL or knock down of BAX, BIM, BAD or apoptosis inducing factor (AIF) protected tumor cells. The drug combination increased AIF : HSP70 co-localization in the cytosol but this event did not prevent AIF : eIF3A association in the nucleus.

## INTRODUCTION

Immune cell activation in general and particularly during rheumatoid arthritis progression requires signaling by Janus kinases (JAK1, JAK2, JAK3). Thus drug companies, attacking these kinases as drugable targets, have developed several FDA approved agents in the hope of reducing the negative sequelae of arthritis as well as myelo-proliferative disorders: Jakafi and Xeljanz [1-4]. Jakafi (ruxolitinib) inhibits JAK1 and JAK2 whereas Xeljanz (tofacitinib) inhibits JAK3 and to a lesser extent JAK1. In the field of cancer research and therapy ruxolitinib has been used, logically based its immune cell actions, in the treatment of myelo-proliferative disorders, myelogenous neoplasms and auto-immune diseases such as psoriasis [5]. The Janus kinases phosphorylate Signal Transducers and Activators of Transcription (STAT) transcription factors on tyrosine resulting in factor dimerization and nuclear localization, and eventually activation of various target genes [6-10]. Thus mutated active forms of Janus kinases or the actions of mutated activated growth factor receptors through autocrine loops cause constitutive activation of the STAT1 / STAT3 / STAT5 transcription factors that promote the malignant phenotype. Growth factor receptors such as ERBB1 and c-MET also have been shown to phosphorylate STAT factors on tyrosine residues thereby promoting dimerization and activation [11, 12]. Cyto-protective genes activated by STAT transcription factors are many and include those coding for: anti-apoptotic genes such as MCL-1, BCL-XL, BCL-2, survivin, HSP90, HSP70; proliferation regulatory genes such as Cyclin D1, Cyclin B, c-Jun, c-Fos; and angiogenesis promoting genes such as HIF1α, and growth factors such as IL-6, FGF, EGF and VEGF [13-20].

It is well known that in the majority of tumor cell isolates from all malignancies, i.e. cells which are generally not addicted to a specific single driving oncogene, that in order to kill the tumor cell effectively *in vitro* and *in vivo* requires the combinatorial use of two or more modulators of signal transduction pathways. For example, published studies from this laboratory combining [MEK1/2 inhibitors + CHK1 inhibitors]; [sorafenib / regorafenib + PI3K/AKT inhibitors]; [sorafenib/regorafenib + ERBB1/2 inhibitors]; [PARP1 inhibitors + CHK1 inhibitors]; [SRC family inhibitors + CHK1 inhibitors]; [ERBB1/2 inhibitors + CDK inhibitors]; and [HSP90 inhibitors + MEK1/2 inhibitors] are a good illustration of this dual pathway inhibition to kill concept [21-27].

More recent studies from this laboratory have extended the dual pathway inhibition killing concept by the use of multiplex assays on drug treated tumors which permit analyses of plasma cytokine levels and the activity status of multiple signal transduction parameters in tumors / tumor cells surviving the dual pathway inhibition treatment. For example, in 2011 we published that the drugs pemetrexed and sorafenib interacted in a synergistic fashion to kill tumor cells *in vitro* and *in vivo* and recently very encouraging data from a phase I trial combining these agents was presented at the 2015 ASCO meeting (NCT01450384). Based on multiplex assays of plasma and tumor material from additional rodent studies we discovered that [pemetrexed + sorafenib] treatment caused a compensatory activation of ERBB1/2 in the tumor cells surviving two drug treatment. And, *in vitro* and *in vivo*, the combination of an ERBB1/2 inhibitor with [pemetrexed + sorafenib] significantly reduced tumor cell viability and tumor growth [28]. Very recently a new phase I trial has opened combining [regorafenib + sildenafil] for all solid tumor patients (NCT02466802). Again, based on multiplex assays of plasma and tumor material from additional rodent studies we discovered that [regorafenib + sildenafil] treatment caused a compensatory activation of AKT, with phosphorylation downstream of GSK3, which correlated with increased plasma levels of bFGF and PDGFbb. Inhibition of PI3K/AKT signaling strongly enhanced [regorafenib + sildenafil] lethality across multiple tumor cell lines [29]. This data is also consistent with the findings in one of our prior studies combining sorafenib/regorafenib with PI3K/AKT inhibitors.

These studies were performed to determine whether the myelo-proliferative disease drug ruxolitinib could be repurposed for use in cancer therapy, specifically whether it would interact with the multiple sclerosis drug dimethyl-fumarate (DMF). The *in vitro* studies in the present manuscript use ruxolitinib at a concentration of 2.5 μM or less to reflect the probable safe achievable level of bioactive drug in a patient.

## RESULTS

**Figure 1:**
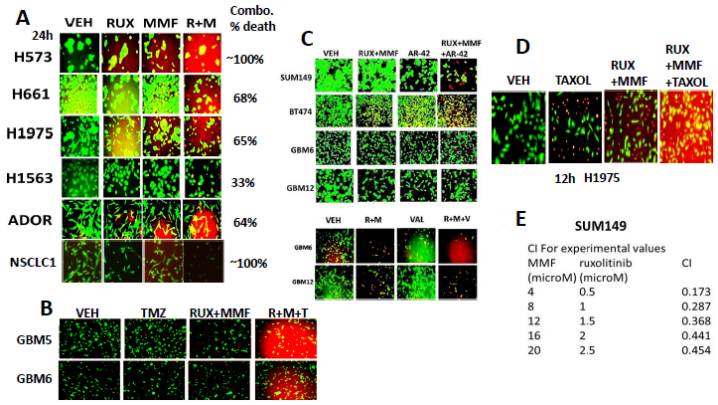
Ruxolitinib synergizes with MMF to kill brain, lung and triple negative breast cancer cells **A.** Non-small cell lung cancer cells were treated with vehicle control, ruxolitinib-phosphate (2.5 μM), MMF (5.0 μM) or the drugs in combination. Twenty four h later cell viability was assessed using a live/dead assay in a Hermes WiScan microscope at 10X magnification (*n* = 3 +/− SEM). **B.** GBM5 and GBM6 cells were treated with vehicle control, Temozolomide (TMZ, 50 nM), [ruxolitinib (1 μM) + MMF (5 μM)], or the three drugs in combination. Twelve hours later, cells were isolated and processed. Cell viability was assessed using a live/dead assay in a Hermes WiScan microscope at 10X magnification (*n* = 3 +/− SEM). **C.** *Upper*: GBM6, GBM12, SUM149 and BT474 cells GBM6, GBM12 and SUM149 cells were treated for 12h with vehicle control or with ruxolitinib (1.0 μM) and MMF (5.0 μM) in the presence of vehicle control or with the HDAC inhibitor AR-42 (0.3 μM). Twelve h after drug exposure cell viability was assessed using a live/dead assay in a Hermes WiScan microscope at 10X magnification. *Lower*; GBM6 and GBM12 cells were treated for 12h with vehicle control or with ruxolitinib (1.0 μM) and MMF (5.0 μM) in the presence of vehicle control or with the HDAC inhibitor Sodium valproate (0.75 μM). Twelve h after drug exposure cell viability was assessed using a live/dead assay in a Hermes WiScan microscope at 10X magnification. **D.** H1975 cells were treated with vehicle control, [ruxolitinib (1.0 μM) and MMF (5.0 μM), paclitaxel (10 nM) or the drugs in combination. Twelve h after drug exposure cell viability was assessed using a live/dead assay in a Hermes WiScan microscope at 10X magnification. **E.** SUM149 cells were plated (250-1,000) cells per well of a six well plate and 12h after plating were treated with vehicle, ruxolitinib (0.5-2.5 μM), MMF (4-20 μM) or in combination at a constant ratio for 24h, as indicated. The media was removed, cells were washed with drug free media, and the cells cultured for another 10 days in drug free media. Cell colonies were fixed, stained and groups of cells > 50 were counted as colonies. The combination index (CI) for synergy was calculated using the Calcusyn for Windows program using the Cho and Tallalay Method (*n* = 2; 12 individual wells per data point +/− SEM). A combination index of less than 0.70 indicates a strong level of tumor-killing synergy between the drugs.

All prior publications examining the biological actions DMF have used the drug at > > 15 μM which is above the safe physiologically achievable plasma level of the actual biologically active *in vivo* break-down product of DMF, mono-methyl fumarate MMF, and as a consequence the key target(s) of *MMF* in cells, transformed or otherwise, are presently unknown. For example, at 5 μM MMF, the *in vitro* changes in expression of a previously claimed DMF target, *Nrf2*, are almost unperceivable (data not shown). Initial studies determined whether MMF interacted with the JAK1/2 inhibitor ruxolitinib to kill lung cancer cells. Established lung cancer cell lines and the July 2015 PDX model ADOR and January 2016 PDX model NSCLC1 were killed by the combination of ruxolitinib and MMF (Figure 1A). [Ruxolitinib + MMF] killed GBM5 and GBM6 cells and enhanced the killing potential of Temozolomide (Figure 1B). The killing power of histone deacetylase inhibitors and of paclitaxel were also enhanced by [ruxolitinib + MMF] (Figure 1C and 1D).

**Figure 2:**
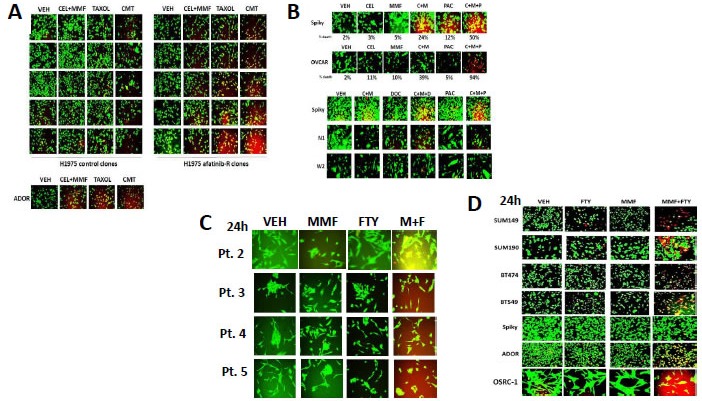
MMF can be used in combination with many different FDA approved drugs to kill tumor cells **A.** H1975 NSCLC cells (5 vehicle control clones; 5 afatinib resistant clones) were treated with vehicle control; paclitaxel (taxol, 10 nM); [MMF 5 μM + celecoxib 5 μM]; or the drugs in combination for 24h. Twenty four hours after these treatments / irradiation, cells were isolated and processed. Cell viability was assessed using a live/dead assay in a Hermes WiScan microscope at 10X magnification. **B.** Ovarian cancer cells (OVCAR, Spiky, N1, W2) were treated with vehicle control; paclitaxel (taxol, 10 nM) or docetaxol (10 nM); [MMF 5 μM + celecoxib 5 μM]; or the drugs in combination for 24h. Twenty four hours after these treatments / irradiation, cells were isolated and processed. Cell viability was assessed using a live/dead assay in a Hermes WiScan microscope at 10X magnification. **C.** Four primary PDX isolates of primary human glioblastoma were treated with vehicle control, MMF (5 μM), FTY720 (100 nM) or the drugs in combination. Twenty four hours after these treatments cells were isolated and processed. Cell viability was assessed using a live/dead assay in a Hermes WiScan microscope at 10X magnification. **D.** Established mammary carcinoma cell lines (SUM149, SUM190, BT474, BT549) and PDX isolates of human cancer (Spiky - ovarian; ADOR - NSCLC; OSCR-1 - osteosarcoma) were treated with vehicle control, MMF (5 μM), FTY720 (100 nM) or the drugs in combination. Twenty four hours after these treatments cells were isolated and processed. Cell viability was assessed using a live/dead assay in a Hermes WiScan microscope at 10X magnification.

The NSAID drug celecoxib has been investigated in the Dent laboratory as a possible anti-cancer agent in combination with a range of drugs. Celecoxib enhanced the killing power of MMF in non-small cell lung cancer cells that express a double mutated active ERBB1 protein (the H1975 cell line) (Figure 2A). The ability of [MMF + celecoxib] treatment to kill H1975 cells and to sensitize these cells to standard of care Taxane drugs was increased in afatinib resistant H1975 cells (5 control; 5 resistant clones shown). In the PDX tumor cell isolate ADOR (NSCLC) the cell isolate was very effectively killed by either [celecoxib + MMF] or by paclitaxel (Figure 2A, lower). In the PDX isolates from ovarian cancer (Spiky, N1, W2) [celecoxib + MMF] to a variable extent enhanced the killing potential of docetaxel and paclitaxel (Figure 2B). The established OVCAR cell line was almost completely killed by the combination of [celecoxib + MMF + paclitaxel]. In addition to MMF, another drug has recently been approved for the treatment of remittent relapsing multiple sclerosis: FTY720 (Fingolimod, Gilenya). MMF and FTY720 interacted to kill multiple fresh PDX models of glioblastoma (Figure 2C). FTY720 and MMF also combined to kill breast cancer cells and PDX models of ovarian cancer, lung cancer and a November 2015 PDX model of osteo-sarcoma (Figure 2D).

**Figure 3:**
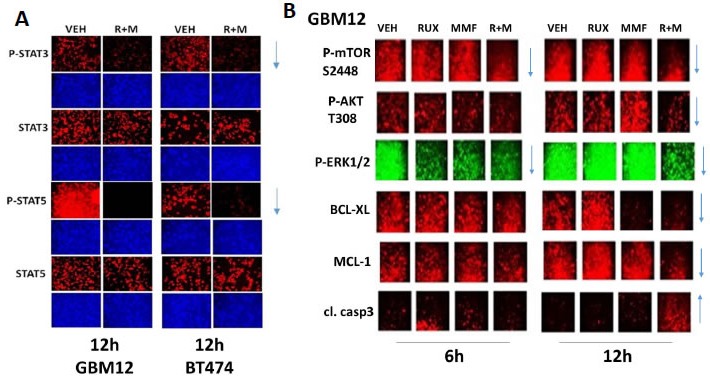
[MMF + Ruxolitinib] interact to cause a prolonged suppression of STAT3, STAT5, ERK1/2, AKT and mTOR phosphorylation **A.**.-**B.** BT474 and GBM12 cells were treated with vehicle control, ruxolitinib (1.0 μM), MMF (5.0 μM) or the drugs in combination for 6h and for 12h, as indicated. At each time point cells were fixed in place and permeabilized using 0.5% Triton X100. Immuno-fluorescence was performed to detect the phosphorylation levels of STAT3, STAT5, ERK1/2, AKT and mTOR, as well as the expression of BCL-XL and MCL-1 and the cleavage status of caspase 3. The arrows show the increase or decrease in expression/function of each protein at the 6h and 12h time points (*n* = 3 +/− SEM).

**Figure 4:**
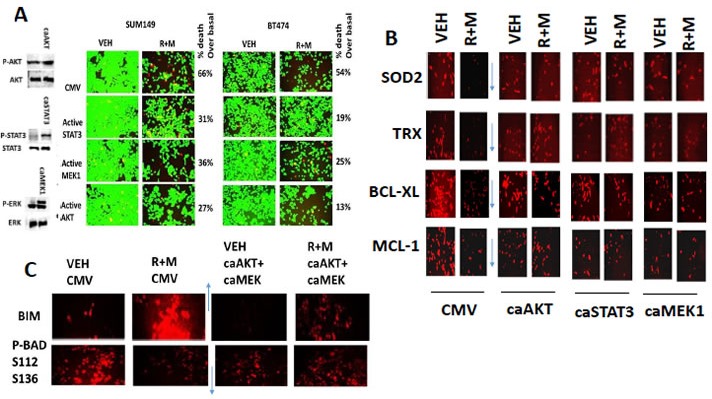
Activation of STAT3, AKT or MEK1 protects cells from [ruxolitinib + MMF], maintains SOD2, TRX, BCL-XL and MCL-1 expression and prevents expression/activation of BIM and BAD **A.** SUM149 and BT474 cells were transfected with an empty vector plasmid (CMV) or with plasmids to express: activated STAT3; activated AKT; or activated MEK1. Twenty four h after transfection cells were treated with vehicle control or with ruxolitinib (1.0 μM) and MMF (5.0 μM) in combination for 24h. Twenty four h later cell viability was assessed using a live/dead assay in a Hermes WiScan microscope at 10X magnification (*n* = 3 +/− SEM). **B.** SUM149 cells were transfected with an empty vector plasmid (CMV) or with plasmids to express: activated STAT3; activated AKT; or activated MEK1. Twenty four h after transfection cells were treated with vehicle control or with ruxolitinib (1.0 μM) and MMF (5.0 μM) in combination for 12h. Cells were fixed in place and permeabilized using 0.5% Triton X100. Immuno-fluorescence was performed to detect the expression of SOD2, TRX, BCL-XL and MCL-1 at 10X magnification in the Hermes WiScan machine (*n* = 3 +/− SEM). **C.** SUM149 cells were transfected with an empty vector plasmid or with plasmids together to express activated AKT and to express activated MEK1. Twenty four h after transfection cells were treated with vehicle control or with ruxolitinib (1.0 μM) and MMF (5.0 μM) in combination for 12h. Cells were fixed in place and permeabilized using 0.5% Triton X100. Immuno-fluorescence was performed to detect the expression of BIM and the S112 S136 phosphorylation of BAD (*n* = 3 +/− SEM).

Ruxolitinib and MMF have the potential to modulate the functions and activities of many intracellular signal transduction pathways, and we next explored the impact our ruxolitinib based drug combination had on cell signaling processes. In mammary and brain cancer cells [MMF + ruxolitinib] significantly inhibited the phosphorylation of STAT3 and STAT5 after 12h of treatment by > 50%, as would be expected based on the declared kinase specificity of ruxolitinib (Figure 3A, *p* < 0.05). [Ruxolitinib + MMF] only transiently inhibited p65 NFκB phosphorylation at 6h after treatment but did cause in combination together a significantly more prolonged > 50% inhibition of ERK1/2, AKT and mTOR activity (Figure 3B, not shown, *p* < 0.05). Both ruxolitinib and MMF as single agents caused activation of JNK1/2, with no obvious positive or negative interaction between the drugs (data not shown). The drug combination significantly reduced expression of the anti-apoptotic mitochondrial and endoplasmic reticulum protective proteins BCL-XL and MCL-1 after 12h of treatment by > 50%, which correlated with increased pro-caspase 3 cleavage, i.e. activation of pro-caspase 3 (Figure 3B, *p* < 0.05). In SUM149 triple negative breast cancer cells and in BT474 ERBB2+ breast cancer cells expression of an activated form of STAT3; an activated form of AKT; or an activated form of MEK1 inhibited the lethality of [ruxolitinib + MMF] treatment (Figure 4A, *p* < 0.05). The [ruxolitinib + MMF] drug combination reduced BCL-XL and MCL-1 expression by over 50% and reduced the expression of super-oxide dismutase 2 (SOD2) and thioredoxin (TRX) by over 75% (Figure 4B, *p* < 0.05). Expression of activated STAT3 or activated AKT or activated MEK1 to very similar extents maintained MCL-1, BCL-XL, SOD2 and TRX expression in the face of [ruxolitinib + MMF] exposure. Unlike the other tested proteins, [ruxolitinib + MMF] treatment had very modest effects at inhibiting the expression of the chaperone proteins HSP90 and HSP70 (data not shown). Treatment of SUM149 cells with [ruxolitinib + MMF] increased the protein expression of the toxic BH3 domain protein BIM by > 400% and reduced the phosphorylation of the toxic BH3 domain protein BAD at S112/S136 by > 50% (Figure 4C, *p* < 0.05). Expression of activated forms of AKT and MEK1 together prevented [ruxolitinib + MMF] from increasing BIM levels or causing BAD S112/S136 dephosphorylation.

**Figure 5:**
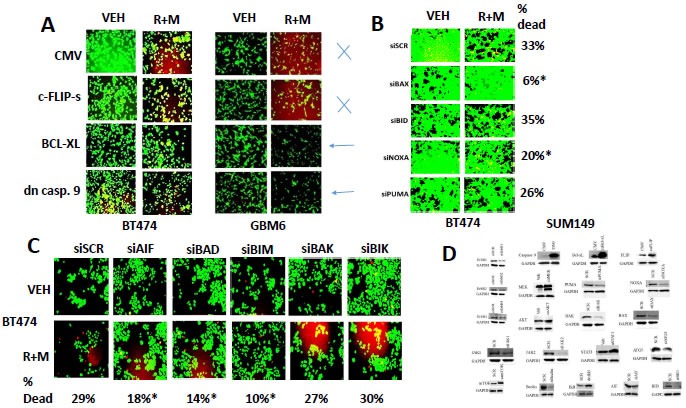
BAX, BAD and BIM signaling play important roles in [ruxolitinib + MMF] lethality **A.** BT474 and GBM6 cells were transfected with an empty vector plasmid or plasmids to express: c-FLIP-s; BCL-XL; or dominant negative caspase 9. Twenty four h after transfection the cells were treated with vehicle control or ruxolitinib (1.0 μM) and MMF (5.0 μM) in combination for 24h. After 24h cell viability was assessed using a live/dead assay in a Hermes WiScan microscope at 10X magnification (*n* = 3 +/− SEM). **B.** BT474 cells were transfected with a scrambled nonsense siRNA control or with siRNA molecules to knock down the expression of: BAX, BID, NOXA, or PUMA. Twenty four h after transfection the cells were treated with vehicle control or ruxolitinib (1.0 μM) and MMF (5.0 μM) in combination for 24h. After 24h cell viability was assessed using a live/dead assay in a Hermes WiScan microscope at 10X magnification (*n* = 3 +/− SEM). **C.** BT474 cells were transfected with a scrambled nonsense siRNA control or with siRNA molecules to knock down the expression of: AIF, BAD, BIM, BAK or BIK. Twenty four h after transfection the cells were treated with vehicle control or ruxolitinib (1.0 μM) and MMF (5.0 μM) in combination for 24h. After 24h cell viability was assessed using a live/dead assay in a Hermes WiScan microscope at 10X magnification (*n* = 3 +/− SEM). **D.** SUM149 cells were transfected with a scrambled siRNA or siRNA molecules to knock down a wide variety of indicated proteins. SUM149 cells were transfected with an empty vector plasmid or with plasmids to express a wide variety of indicated proteins. These images are presented as control data for our knock down and over-expression studies.

Based on our immuno-fluorescence data in Figure 4, caspase 3 was being cleaved (activated) after drug combination treatment, and we next investigated the molecular mechanisms by which [ruxolitinib + MMF] treatment was killing tumor cells. Inhibition of caspase 8 / death receptor signaling by over-expression of c-FLIP-s did not significantly reduce the lethality of [ruxolitinib + lapatinib] treatment (Figure 5A, *p* < 0.05). Over-expression of BCL-XL or to a lesser extent inhibition of caspase signaling downstream of mitochondria by expression of dominant negative caspase 9 significantly reduced drug combination killing by > 50% (Figure 5A, *p* < 0.05). Knock down of the toxic BH3 domain protein BAX and to a much lesser extent NOXA, but not knock down of BID or PUMA, abolished drug combination lethality and the anti-proliferative effect of the drug combination (Figure 5B, *p* < 0.05). Knock down of AIF, BAD and BIM, but not BIK or BAK, also significantly reduced [ruxolitinib + MMF] lethality (Figure 5C, *p* < 0.05). *Nota bene*: compare the protective effects of BAD and BIM knock down in Figure 5 to the regulation of BIM/BAD expression/activity in Figure 4 by [ruxolitinib + MMF] and activated AKT/MEK. Control knock down / over-expression immuno-blots are presented in Figure 5D for SUM149 cells which are near identical to previously published data for BT474 and GBM cells.

**Figure 6:**
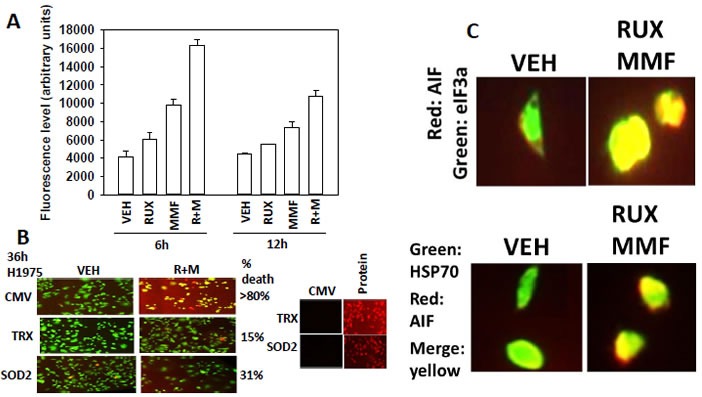
[Ruxolitinib + MMF] exposure increases ROS levels and causes release of AIF into the cytosol and AIF localization in the nucleus **A.** SUM149 cells were treated with vehicle control or with, ruxolitinib (1.0 μM), MMF (5.0 μM)] or in combination for 6h and 12h. Fifteen minutes before each time point cells are incubated with diacetate ester dichlorofluorescin (DCFH), 5 μM. The conversion of DCFH to DCF by reactive oxygen and nitrogen species is measured in sextuplicate in a Vector 3 plate reader (*n* = 3 +/− SEM).**B.** SUM149 cells were transfected with empty vector control (CMV) or with plasmids to express super-oxide dismutase 2 (SOD2) or thioredoxin (TRX). Twenty four h after transfection cells were treated with vehicle control or with, ruxolitinib (1.0 μM), MMF (5.0 μM)] or in combination for 36h. Cell viability was assessed using a live/dead assay in a Hermes WiScan microscope at 10X magnification (*n* = 3 +/− SEM). **C.** Upper: GBM12 cells were treated with vehicle control or with [ruxolitinib (1.0 μM) + MMF (5.0 μM)] for 6h. After 6h cells were fixed in place and permeabilized using 0.5% Triton X100. Immuno-fluorescence was performed to detect the expression of AIF (red fluorescent stain); eIF3A (green fluorescent stain) with images at 60X magnification. Lower: GBM12 cells were treated with vehicle control or with [ruxolitinib (1.0 μM) + MMF (5.0 μM)] for 6h. After 6h cells were fixed in place and permeabilized using 0.5% Triton X100. Immuno-fluorescence was performed to detect the expression of AIF (red fluorescent stain) and HSP70 (green fluorescent stain). The co-localization of AIF with HSP70 was determined by merging the images in Adobe Photoshop CS6 at 9999 dpi.

In Figure 4 we discovered that [ruxolitinib + MMF] treatment decreased expression of the reactive oxygen species de-toxifying enzymes SOD2 and TRX. Thus we next determined whether [ruxolitinib + MMF] treatment altered the production of reactive oxygen species (ROS) in tumor cells and whether the over-expression of SOD2 or TRX could suppress drug combination toxicity. Ruxolitinib and MMF interacted in a greater than additive fashion to increase ROS levels over the 12h following drug combination exposure (Figure 6A). Over-expression of either SOD2 or TRX significantly reduced the lethality of [ruxolitinib + MMF] (Figure 6B, *p* < 0.05). Note: at 2.5X the image intensity of the over-expression data, basal SOD2 and TRX levels are observed. Treatment of cells with [ruxolitinib + MMF] caused the co-localization of apoptosis inducing factor (AIF) with both the cytosolic protein HSP70 and the nucleo-protein eIF3a (Figure 6C). i.e. HSP70 was unable to sequester all of the AIF released from the mitochondrion in the cytoplasm, and thus prevent it from entering the nucleus. Thus [ruxolitinib + MMF] treatment kills tumor cells through prolonged inactivation of multiple upstream protective signaling pathways which leads to lower levels of protective BH3 domain proteins (BCL-XL, MCL-1) and to higher levels of toxic activated BH3 domain proteins (BIM, BAD) which promotes mitochondrial dysfunction and ROS generation. Inactivation of the protective signaling pathways also lowers the expression of the detoxification enzymes SOD2 and TRX which additionally facilitates sustained high levels of toxic ROS, all collectively leading to tumor cell death.

## DISCUSSION

The present studies were undertaken to determine whether the myelo-proliferative disorder medication and JAK1/2 inhibitor ruxolitinib could be re-purposed as a cancer therapeutic against solid tumors. We discovered that ruxolitinib at clinically relevant free drug concentrations synergized with the multiple sclerosis drug MMF to kill a wide variety of solid tumor cell types, particularly breast and brain tumor cells, including those cells expressing mutated active RAS or ERBB1 proteins, or lacking the tumor suppressor PTEN.

Killing by [ruxolitinib + MMF] occurred in a wide variety of tumor cell types, including triple negative breast cancer cells isolated from inflammatory breast cancer patients and multiple PDX models of glioblastoma, and lung cancer. In both BT474 and in SUM149 mammary carcinoma cells, constitutively active forms of STAT3, AKT and MEK1 were each shown to be a key protective molecular signal to suppress [ruxolitinib + MMF] -induced killing. In part, each of these activated proteins was protecting cells because individually they increased the basal expression of the mitochondrial protective proteins MCL-1 and BCL-XL and the reactive oxygen species de-toxifying enzymes SOD2 and TRX. That (MCL-1 and BCL-XL) and (TRX and SOD2) are negatively regulated by [ruxolitinib + MMF] treatment is in agreement with the mode of cell killing; intrinsic pathway activation in parallel with AIF translocation to the nucleus. That any one of three signaling pathways can maintain expression of these proteins in the face of the drug combination argues that it is the ability of [ruxolitinib + MMF] to simultaneously inhibit the STAT3, AKT and MEK-ERK pathways which is crucial in promoting tumor cell killing.

In addition to modulating the expression of anti-apoptotic proteins, [ruxolitinib + MMF] also controlled the expression and phosphorylation of pro-apoptotic toxic BH3 domain proteins. [Ruxolitinib + MMF] treatment increased the expression of BIM and decreased the S112+S136 phosphorylation of BAD within 6h; both events would be predicted to cause mitochondrial dysfunction. Maintained signaling through the ERK1/2 and AKT pathways by molecular interventions prevented the drug combination -induced expression of BIM or the dephosphorylation of BAD. Hence, it is the combination of reduced BCL-XL / MCL-1 expression and enhanced pro-apoptotic signaling through BIM / BAD / BAX which facilitates AIF release into the cytosol.

In addition to the [ruxolitinib + MMF] two drug combination we also determined whether clinically relevant standard of care agents for other cancers could enhance the lethality of the two drug combination. Treatment of breast and brain cancer cells with [MMF + ruxolitinib] for 12h resulted in tumor cell killing; treatment of these tumor cells with [MMF + ruxolitinib] together with either Temozolomide, paclitaxel or with HDAC inhibitors further enhanced the lethality of the initial two drug combination. As the use of sodium valproate as a standard of care agent for glioblastoma in some cohorts of patients predicts for a better survival outcome, e.g. in Dundee, Scotland and Richmond VA USA, the combination of [MMF + ruxolitinib + valproate] also could represent a viable relatively cheap novel approach to treat recurrent GBM.

Over the past 18 months the Dent laboratory has published several manuscripts which demonstrate the usefulness of the NSAID drug and COX2 inhibitor celecoxib, at low clinically relevant concentrations, as a dose-response modifier for other established and novel anti-cancer agents. Unlike the celecoxib derivative drug OSU-03012 (AR12), celecoxib does not inhibit the chaperone ATPase activities of either HSP90 or of HSP70, yet was shown competent to cause an endoplasmic reticulum stress response indicative that the chaperone activities of GRP78 and other chaperone proteins had been disrupted. Celecoxib and MMF interacted to kill tumor cells, including PDX isolates, and furthermore, enhanced the ability of paclitaxel (Taxol) and docetaxel (Taxotere) to kill lung and ovarian cancer cells [30-33]. As mentioned previously, the majority of published studies using MMF, as well as celecoxib, have used both drugs at many times their safe achievable doses in a patient. Further detailed mechanistic studies will be required to understand how celecoxib and MMF interact to kill, and how they in combination enhance the lethality of Taxane drugs against standard of care tumor types.

As noted previously in this manuscript, many published studies using MMF (actually DMF) and ruxolitinib use the drugs at > > 15 μM and as such, the additional novel key targets beyond those described in this manuscript using much lower drug concentrations are unclear. Hence studies beyond the scope of the present paper will be required to more completely understand how [ruxolitinib + MMF] interact to kill and, based on the cost of translation into the clinic, whether [MMF + ruxolitinib] is a drug combination that can translate into the clinic for solid tumor or specifically GBM patients.

## MATERIALS AND METHODS

### Materials

Ruxolitinib phosphate was purchased from Selleckchem (Houston, TX). Trypsin-EDTA, DMEM, RPMI, penicillin-streptomycin were purchased from GIBCOBRL (GIBCOBRL Life Technologies, Grand Island, NY). Cells were purchased from the ATCC and were not further validated beyond that claimed by ATCC. Cells were re-purchased every ∼6 months. Primary human glioblastoma (GBM) cells, developed by Dr. C.D. James when at the Mayo Clinic (Rochester, MN) has been described previously. ADOR non-small cell lung cancer cells are personal a donation from the patient to the Dent laboratory. De novo cisplatin resistant “Spiky” ovarian cancer cells, a patient derived explant (PDX) model, were kindly provided by Dr. Karen Paz (Champions Oncology, NJ). The plasmids to express thioredoxin (TRX) and mutant thioredoxin (mTRX) were a kind gift from Dr. David Gius (Radiobiology Branch, National Cancer Institute, Bethesda, MD). The other plasmids in these studies were purchased from Addgene (Cambridge, MA). Commercially available validated short hairpin RNA molecules to knock down RNA / protein levels were from Qiagen (Valencia, CA) or were supplied by collaborators. Reagents and performance of experimental procedures were described in refs: [21-29, 30, 31, 32].

### Methods

#### Culture and *in vitro* exposure of cells to drugs

All cell lines were cultured at 37°C (5% (v/v CO_2_) *in vitro* using RPMI supplemented with dialyzed 5% (v/v) fetal calf serum and 10% (v/v) Non-essential amino acids. *In vitro* drug treatments were from 100 mM stock solutions of each drug and the maximal concentration of Vehicle (DMSO) in media was 0.02% (v/v). Cells were not cultured in reduced serum media during any study in this manuscript.

#### Transfection of cells with siRNA or with plasmids

#### For plasmids

Cells were plated and 24h after plating, transfected. Plasmids expressing a specific mRNA (or siRNA) or appropriate vector control plasmid DNA was diluted in 50μl serum-free and antibiotic-free medium (1 portion for each sample). Concurrently, 2μl Lipofectamine 2000 (Invitrogen), was diluted into 50μl of serum-free and antibiotic-free medium (1 portion for each sample). Diluted DNA was added to the diluted Lipofectamine 2000 for each sample and incubated at room temperature for 30 min. This mixture was added to each well / dish of cells containing 200μl serum-free and antibiotic-free medium for a total volume of 300 μl, and the cells were incubated for 4 h at 37°C. An equal volume of 2x medium was then added to each well. Cells were incubated for 24h, then treated with drugs.

#### Transfection for siRNA

Cells from a fresh culture growing in log phase as described above, and 24h after plating transfected. Prior to transfection, the medium was aspirated and serum-free medium was added to each plate. For transfection, 10 nM of the annealed siRNA, the positive sense control doubled stranded siRNA targeting GAPDH or the negative control (a “scrambled” sequence with no significant homology to any known gene sequences from mouse, rat or human cell lines) were used. Ten nM siRNA (scrambled or experimental) was diluted in serum-free media. Four μl Hiperfect (Qiagen) was added to this mixture and the solution was mixed by pipetting up and down several times. This solution was incubated at room temp for 10 min, then added drop-wise to each dish. The medium in each dish was swirled gently to mix, then incubated at 37°C for 2h. Serum-containing medium was added to each plate, and cells were incubated at 37°C for 24h before then treated with drugs (0-24h). Additional immuno-fluorescence / live-dead analyses were performed at the indicated time points.

### Detection of cell viability, protein expression and protein phosphorylation by immuno-fluorescence using a hermes wiScan machine

http://www.idea-bio.com/, Cells (4 × 10^3^) are plated into each well of a 96 well plate, and cells permitted to attach and grow for the next 18h. Based on the experiment, after 18h, cells are then either genetically manipulated, or are treated with drugs. For genetic manipulation, cells are transfected with plasmids or siRNA molecules and incubated for an additional 24h. Cells are treated with vehicle control or with drugs at the indicated final concentrations, alone or in combination. Cells are then isolated for processing at various times following drug exposure. The 96 well plate is centrifuged / cyto-spun to associate dead cells (for live-dead assays) with the base of each well. For live dead assays, after centrifugation, the media is removed and cells treated with live-dead reagent (Thermo Fisher Scientific, Waltham MA) and after 10 min this is removed and the cells in each well are visualized in the Hermes instrument at 10X magnification. Green cells = viable; yellow/red cells = dying/dead. The numbers of viable and dead cells were counted manually from three images taken from each well combined with data from another two wells of separately treated cells (i.e. the data is the mean cell dead from 9 data points from three separate exposures). For immuno-fluorescence studies, after centrifugation, the media is removed and cells are fixed in place and permeabilized using ice cold PBS containing 0.4% paraformaldehyde and 0.5% Triton X-100. After 30 min the cells are washed three times with ice cold PBS and cells are pre-blocked with rat serum for 3h. Cells are then incubated with a primary antibody to detect the expression/phosphorylation of a protein (usually at 1:100 dilution from a commercial vendor) overnight at 37°C. Cells are washed three times with PBS followed by application of the secondary antibody containing an associated fluorescent red or green chemical tag. After 3h of incubation the antibody is removed and the cells washed again. The cells are visualized at either 10X or 60X in the Hermes machine for imaging assessments. All immunofluorescent images for each individual protein / phospho-protein are taken using the identical machine settings so that the levels of signal in each image can be directly compared to the level of signal in the cells treated with drugs. Similarly, for presentation, the enhancement of image brightness/contrast using PhotoShop CS6 is simultaneously performed for each individual set of protein/phospho-protein to permit direct comparison of the image intensity between treatments.

For SDS-PAGE and immunoblotting, cells were plated at 5 × 10^5^ cells/cm^2^ and treated with drugs at the indicated concentrations and after the indicated time of treatment, lysed in whole-cell lysis buffer (0.5 M Tris-HCl, pH 6.8, 2% SDS, 10% glycerol, 1% *β*-mercaptoethanol, 0.02% bromophenol blue), and the samples were boiled for 30 minutes. The boiled samples were loaded onto 10-14% SDS-PAGE and electrophoresis was run overnight (10-100 *μ*g/lane based on the gel size). Proteins were electrophoretically transferred onto 0.22-*μ*m nitrocellulose, and immunoblotted with various primary antibodies against different proteins. Antibodies used include: All immunoblots were initially visualized at 75 dpi using an Odyssey infrared imager (Li-Cor, Lincoln, NE), then processed at 9999 dpi using Adobe Photoshop CS6. For presentation, immunoblots were digitally assessed using the provided Odyssey imager software. Images have their color removed and labeled figures generated in Microsoft PowerPoint.

### Animal studies

For studies to generate afatinib resistant H1975 cells, pre-existing tumors as above were treated with afatinib (50 mg/kg) BID for 4 days. This reduced tumor volume of all clones to 0 for approximately 7 days after which tumors began to slowly re-grow. Recurrent tumors were isolated on Day 25, portions were snap-frozen or were digested to release individual tumor cells, and cells from each tumor clone maintained separately. Of significant note for clonal characterization, the isolated afatinib treated tumor cells were only growth inhibited by afatinib *in vitro* with daily supplementation at concentrations > > 2 μM, and as such these cells were routinely passaged in a pulsatile fashion between experiments in growth media containing only 1 μM afatinib to maintain the phenotype but not to promote further selective pressure on drug resistance.

### Assessment of ROS generation

Cancer cells were plated in 96 well plates. Cells were treated with the drugs and 15 min prior to the indicated time point the media was removed and cells incubated with diacetate dihydro-DCF (5 μM). Fluorescence measurements were obtained 15 minutes after DCFH addition with a Vector 3 plate reader. Data are presented corrected for basal fluorescence of vehicle-treated cells at each time point and expressed as the arbitrary units provided by the plate reader / the increase in ROS levels.

### Data analysis

Comparison of the effects of various treatments was performed using one way analysis of variance and a two tailed Student's *t*-test. Statistical examination of *in vivo* animal survival data utilized log rank statistical analyses between the different treatment groups. Differences with a *p*-value of < 0.05 were considered statistically significant. Experiments shown are the means of multiple individual points from multiple experiments (± SEM).

## Abbreviations

ERK: extracellular regulated kinase
MEK: mitogen activated extracellular regulated kinase
PI3K: phosphatidyl inositol 3 kinase
ca: constitutively active
dn: dominant negative
ER: endoplasmic reticulum
mTOR: mammalian target of rapamycin
JAK: Janus Kinase
STAT: Signal Transducers and Activators of Transcription
MAPK: mitogen activated protein kinase
PTEN: phosphatase and tensin homologue on chromosome ten
ROS: reactive oxygen species
CMV: empty vector plasmid or virus
si: small interfering
SCR: scrambled
IP: immunoprecipitation
Ad: adenovirus
VEH: vehicle
